# Magnesii Sulphas

**Published:** 1890-04

**Authors:** Will. H. Whitslar

**Affiliations:** Youngstown, O.


### Magnesii Sulphas.

BY WILL. H. WHITSLAR, D.D.S., YOUNGSTOWN, O.

Sulphate of magnesium, or as it is ordinarily known as Epsom Salt, and very commonly called "salts," is one of the most powerful and reliable medicaments that the dentist can use in certain cases.

In these days of antiseptic treatment of root-canals our cases of failure are few compared to those previous to the introduction of germicides and the rubber dam. But it happens even to the best operator to have introduced to him unexpectedly, some blustering winter morning, perhaps, a personal observation of a character that—well, we wish it didn't happen, and it would be far more pleasant to us if some other dentist had the situation to deal with.

Now it happens to be a patient for the loss of whom we might lose in his family and others whom they come in contact with, reputation, fees, personal friendship, every thing, in fact, for the subterfuge, "You have taken cold and it has settled in your tooth," is not tenable in the vast majority of cases. We must do something and do it immediately. But, why the loss of a good patron when the good old-fashioned remedies are at hand.

The case is about like this :—The conditions are, great arterial tension nearing congestion, and already congestion beginning, swelling of the parts plainly significant, and altogether we have an incipient abscess forming.

Oft times painting the gums with equal parts of tinctures of iodine and aconite rad. may suffice. A capsicum bag may also be of avail. Experience teaches us to recognize certain appearances that we would call "angry," and lines of determination, so to speak, show that decisive action must be taken. What will be the proper course to pursue? Iodine and aconite? Capsicum bag? Dover's powder? No! Scarification? No! No pus to evacuate yet—only pain results and unnessarily given.

The most reliable remedy is Epsom salts! given at the proper time, it is the best remedy that can be used to abort the trouble. The prime object to be obtained is to relieve arterial tension which the magnesia does by causing watery evacuations caused by the tissues being deprived of water. No patient will object to this treatment when it is explained what the conditions are about the tooth, and the result if allowed to proceed, and how the magnesia acts upon the system. It will seem incredulous to the patient that the trouble will be ended by this means, but having gained their confidence, and desiring relief, the victory is won—if you prescribe the remedy in time. At a certain period no remedy will abort an abscess. It is for our judgment to decide *when* to give. The next question is *how* to give the magnesia, which has rather a bitter taste. Many will take it in simple solution, the average dose being about a tablespoonful of the salt in ½ glass to a full glass of water. To make more palatable use carbonic acid water commonly called at the soda-water fountain "plain soda" with the salt and enough lemon syrup to flavor. Always use Epsom salt; not the Rochelle salt; the case is imperative, so use most effective remedy.

---

A cablegram states that much indignation has been aroused in the two hundred American medical graduates in Berlin, because the authorities at the university have determined not to recognize as valid any diplomas issued by medical colleges in the United States.

